# A hidden threat: incidental diagnosis of goblet cell carcinoma of the appendix

**DOI:** 10.1093/jscr/rjaf827

**Published:** 2025-10-28

**Authors:** Marco A Romo, Shivam Pandya, Makrouhi A Kademian

**Affiliations:** Department of General Surgery, Los Robles Regional Medical Center, 215 W Janss Road, Thousand Oaks, CA 91360, United States; Department of General Surgery, Los Robles Regional Medical Center, 215 W Janss Road, Thousand Oaks, CA 91360, United States; Department of General Surgery, Los Robles Regional Medical Center, 215 W Janss Road, Thousand Oaks, CA 91360, United States

**Keywords:** goblet, carcinoma, appendix, pathology

## Abstract

Goblet cell carcinoma (GCC) of the appendix is an exceptionally rare and distinct appendiceal neoplasm characterized by mixed features of both neuroendocrine tumors and adenocarcinomas. Its insidious presentation, frequently mimicking more common conditions like acute appendicitis, contributes to diagnostic challenges and highlights its rarity. We present the case of a 70-year-old female who presented to clinic with right-sided abdominal aching and intermittent nausea. Workup revealed colon nodule and she was taken to the operating room for colon resection. An incidental appendectomy was performed given the abnormal appearance of her appendix intraoperatively. Pathology revealed high grade GCC of the appendix, and she was taken back for a laparoscopic right hemicolectomy during the same admission. This case underscores the profound rarity of appendiceal GCC, with an estimated incidence of 0.01 to 0.05 per 100 000 individuals. The diagnostic ambiguity necessitates a high index of suspicion and meticulous pathological review.

## Introduction

Appendiceal neoplasms are uncommon, accounting for <1% of all gastrointestinal cancers. Among these rare tumors, goblet cell carcinoma (GCC) of the appendix stands out as a particularly unique and infrequently encountered entity. First described in 1969, GCC is characterized by a distinctive biphasic differentiation, exhibiting features of both neuroendocrine tumors and conventional adenocarcinomas, predominantly composed of mucin-producing goblet-like cells [1, 2].

The true incidence of appendiceal GCC is remarkably low, estimated to be between 0.01 and 0.05 cases per 100 000 individuals per year, making it a truly rare diagnosis [3]. It is found incidentally in <1% of all appendectomy specimens, often in patients presenting with symptoms indistinguishable from acute appendicitis [4]. This rarity, coupled with its non-specific clinical presentation, poses significant diagnostic challenges for clinicians and pathologists alike. Understanding the unique biological behavior and appropriate management strategies for GCC is crucial, despite its infrequency. This report details a case of incidentally discovered appendiceal GCC, highlighting its rarity and the importance of thorough pathological evaluation.

## Case report

**Figure 1:**
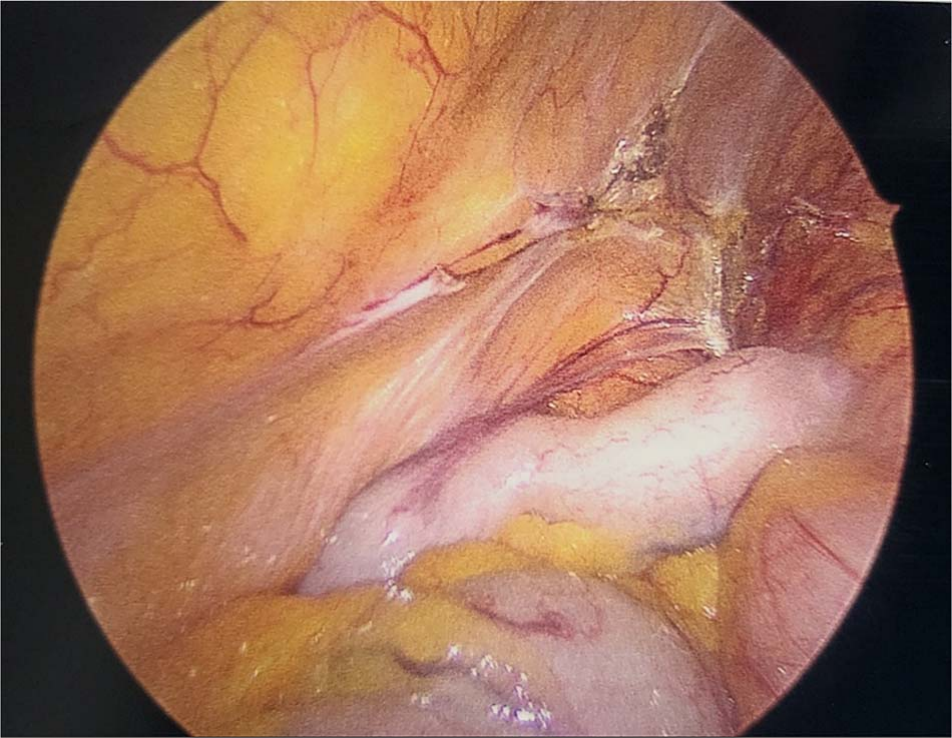
Intraoperative image of appendix during initial index procedure.

A 70-year-old female presented to our clinic with right-sided abdominal aching and intermittent nausea. She had an extensive workup by her gastroenterologist and was ultimately found to have a 2 cm sessile nodule at the hepatic flexure and cholelithiasis with gallbladder thickening. She was taken to the operating theater for an elective laparoscopic partial colon resection with cholecystectomy. At the end of the case, the appendix was noted to be abnormal and enlarged (Fig. 1). Specifically, the tip of the appendix was thick and much of the body was ectatic and thick as well. Out of concern for malignancy, the decision was made to perform an incidental appendectomy. The patient tolerated the procedure well and recovered appropriately in the postoperative period.

Surgical specimen pathology results were released on post-operative day two. Within the appendiceal wall, nests and cords of atypical goblet-like cells were identified, infiltrating the subserosa and muscularis propria. These cells contained abundant intracellular mucin and exhibited high grade nuclear atypia. Immunohistochemical staining confirmed the dual differentiation, showing positivity for both neuroendocrine markers (e.g. chromogranin A, synaptophysin) and epithelial markers (e.g. CK20, SATB2). Based on these characteristic features, a diagnosis of GCC of the appendix was made. The tumor was staged as pT3N0. The tumor was located in the mid appendix and measured 3 cm in size. All margins and nodes included with specimen were negative for invasive carcinoma.

After the pathology results were reviewed with the patient and her family, the decision was made to proceed back to the operating theater 3 days after her initial surgery for formal right hemicolectomy for oncologic resection and staging. During this operation, mild adhesions were encountered in light of recent surgery, but there was no carcinomatosis noted. A laparoscopic right hemicolectomy was performed without issue. The patient recovered appropriately post-operatively and was discharged on post-operative day three with instruction to follow up in outpatient clinic.

Soon after discharge, final pathology of the surgical specimen from the right hemicolectomy revealed no evidence of malignancy or dysplasia in the colon, small bowel, or any of the 13 lymph nodes taken. The stage of her GCC was determined to be T3N0M0. After in depth discussion at multidisciplinary institutional tumor board, since the malignancy had not spread and complete resection was achieved, the recommendation was made for close surveillance without adjuvant chemotherapy.

## Discussion

This case exemplifies the challenges associated with diagnosing appendiceal GCC. With an incidence of <1 per 100 000 population, GCC is an infrequent encounter for most clinicians and pathologists [3]. Although not the case in our scenario, GCC is often an incidental finding during appendectomy for presumed appendicitis [4, 5]. This highlights the critical importance of submitting all appendectomy specimens for comprehensive histopathological examination, regardless of the intraoperative appearance.

The unique histopathological features of GCC, combining elements of both neuroendocrine and glandular differentiation, often lead to diagnostic ambiguity. This tumor has been a source of confusion regarding its classification and nomenclature, leading to terms like “adenocarcinoid” or “goblet cell carcinoid”. However, current understanding emphasizes its more aggressive behavior, akin to adenocarcinoma, hence the preferred term “goblet cell carcinoma” [1, 6]. The infiltrative growth pattern of mucin-filled cells, often forming small nests or cords within the appendiceal wall, is characteristic and requires careful differentiation from typical carcinoids or conventional mucinous adenocarcinomas. Immunohistochemistry has an integral role in confirming the diagnosis by demonstrating the dual lineage.

The prognosis of GCC is generally considered intermediate between that of typical appendiceal carcinoids (which are usually indolent) and conventional appendiceal adenocarcinomas (which are often more aggressive) [5]. Prognostic factors include tumor stage, grade, presence of lymph node metastasis, and peritoneal dissemination. In our patient, the early stage (T3N0M0) and complete resection were favorable prognostic indicators.

Management of appendiceal GCC primarily involves surgical resection. For early-stage, localized disease, a simple appendectomy may be sufficient if margins are clear and there is no evidence of nodal or distant spread. However, for higher-stage tumors (e.g. T2 or greater, positive margins, nodal involvement, or poorly differentiated histology), a right hemicolectomy is often recommended to ensure adequate oncologic clearance and regional lymphadenectomy [7]. The role of adjuvant chemotherapy is still not clearly defined due to the rarity of the disease and the lack of large prospective studies, but it may be considered for advanced or high-risk cases, often following protocols for colorectal adenocarcinoma. Additionally, cytoreductive surgery and hyperthermic intraperitoneal chemotherapy may be indicated in cases where the cancer has spread within the abdomen. Lastly, long-term surveillance is crucial for all patients with GCC due to the potential for recurrence, particularly peritoneal dissemination.

Appendiceal GCC remains a profoundly rare and often unsuspected diagnosis. This case report underscores the necessity for clinicians to maintain a high index of suspicion and, more importantly, for pathologists to perform meticulous and comprehensive histopathological examination of all appendectomy specimens. Accurate diagnosis is paramount for guiding appropriate surgical management and subsequent surveillance, ultimately improving patient outcomes for this unique and uncommon appendiceal malignancy.
